# Monitoring Insulin Aggregation via Capillary Electrophoresis

**DOI:** 10.3390/ijms12129369

**Published:** 2011-12-14

**Authors:** Elizabeth Pryor, Joseph A. Kotarek, Melissa A. Moss, Christa N. Hestekin

**Affiliations:** 1 Ralph E. Martin Department of Chemical Engineering, 3202 Bell Engineering Center, University of Arkansas, Fayetteville, AR 72701, USA; E-Mail: npryor@uark.edu (E.P.); 2 Department of Chemical Engineering, 2C02 Swearingen Engineering Center, University of South Carolina, Columbia, SC 29208, USA; E-Mail: mossme@cec.sc.edu

**Keywords:** capillary electrophoresis, ultraviolet absorbance, laser induced fluorescence, thioflavin T, insulin, oligomer, amyloid

## Abstract

Early stages of insulin aggregation, which involve the transient formation of oligomeric aggregates, are an important aspect in the progression of Type II diabetes and in the quality control of pharmaceutical insulin production. This study is the first to utilize capillary electrophoresis (CE) with ultraviolet (UV) detection to monitor insulin oligomer formation at pH 8.0 and physiological ionic strength. The lag time to formation of the first detected species in the aggregation process was evaluated by UV-CE and thioflavin T (ThT) binding for salt concentrations from 100 mM to 250 mM. UV-CE had a significantly shorter (5–8 h) lag time than ThT binding (15–19 h). In addition, the lag time to detection of the first aggregated species via UV-CE was unaffected by salt concentration, while a trend toward an increased lag time with increased salt concentration was observed with ThT binding. This result indicates that solution ionic strength impacts early stages of aggregation and β-sheet aggregate formation differently. To observe whether CE may be applied for the analysis of biological samples containing low insulin concentrations, the limit of detection using UV and laser induced fluorescence (LIF) detection modes was determined. The limit of detection using LIF-CE, 48.4 pM, was lower than the physiological insulin concentration, verifying the utility of this technique for monitoring biological samples. LIF-CE was subsequently used to analyze the time course for fluorescein isothiocyanate (FITC)-labeled insulin oligomer formation. This study is the first to report that the FITC label prevented incorporation of insulin into oligomers, cautioning against the use of this fluorescent label as a tag for following early stages of insulin aggregation.

## 1. Introduction

Human insulin is a 51-residue protein hormone which stimulates the transport of glucose from blood into cells [1]. *In vivo*, insulin exists as a Zn^2+^ containing hexamer and is stored in the pancreas [2]. Upon dilution in the bloodstream, insulin dissociates rapidly through dimers to biologically active monomers [2]. *In vitro*, insulin exists as a mixture of monomer and oligomers, including dimers and hexamers [3]. Insulin is prone to form amyloid fibrils under various conditions both *in vitro* and *in vivo* [4,5]. It has been postulated that insulin aggregation both *in vitro* and *in vivo* occurs due to the presence of a destabilized monomer that undergoes non-native self-assembly by overcoming the free energy barrier [6–8]. This self-assembly proceeds through the formation of high-order oligomeric species and culminates with the appearance of insoluble fibrillar aggregates. Insulin fibrillization poses a problem for the treatment of Type II diabetes where insulin amyloid deposits have been observed at sites of repeated insulin injection [9–11]. These amyloid deposits are associated with the clinical syndrome, injection-localized amyloidosis [9,10]. It has been proposed that insulin is destabilized in the presence of hydrophobic interfaces such as the solid-aqueous interface of insulin pumps [5,12], leading to its aggregation. The *in vivo* deposition of insulin aggregates can lead to injection site problems for Type II diabetes patients, such as infection, bleeding, bruising, irritation, and inflammation [13]. In addition, insulin fibrillization *in vitro* presents a problem for the quality control of pharmaceutical insulin production [5]. Therefore, it is important to elucidate the molecular mechanisms underlying insulin amyloid fibrillization to improve the treatment of diabetes.

The visualization of oligomers, which appear in the early stages of aggregation, is one key to understanding the molecular mechanisms underlying amyloid formation. Various techniques have been utilized to detect soluble and low-molecular weight oligomeric species formed by amyloid proteins such as atomic force microscopy (AFM) [12,14], light scattering [12,14], hydrogen-deuterium exchange mass spectrometry [15,16], matrix assisted laser desorption ionization mass spectrometry (MALDI-MS) [17,18], electrospray ionization mass spectrometry (ESI-MS) [19], ion mobility mass spectrometry (IM-MS) [20–23], and oligomer specific antibodies [24–26]. A major analytical challenge is developing a technique which is capable of identification, quantification, and characterization of a wide range of amyloid species. Electrophoretic techniques can be used to detect soluble and low-molecular weight oligomeric species and provide a compliment for other traditional techniques. These electrophoretic techniques include sodium dodecyl sulfate polyacrylamide gel electrophoresis (SDS-PAGE) [27–30], Western immunoblotting [27,29,31–37], and capillary electrophoresis (CE) [38–43]. SDS-PAGE is a commonly used technique, but SDS has been reported to accelerate β sheet formation during amyloid aggregation [44,45], to induce and possibly stabilize aggregation [36], and to misrepresent the native species and their assembly [46]. Western blotting necessitates the use of expensive and specific antibodies and can also require a pre-concentration step such as immunoprecipitation [47,48]. In addition, these gel-based methods can produce smears making specific oligomer size determination impossible [29,30]. In contrast, CE provides the ability to inexpensively monitor the aggregation of insulin under native conditions.

Capillary electrophoresis (CE) offers fast and highly efficient separation of molecules with a broad range of properties thereby making it well suited for the analyses of biological samples, which contain different types and sizes of proteins [49]. CE separates proteins based on electrophoretic mobility, which is related to charge, shape, and/or size. Previous studies have demonstrated the utility of CE to detect low concentrations of insulin [50–52] and identify differences in insulin analogs [42]. In this work, we have extended CE to monitor the appearance of insulin oligomers over time when aggregation is carried out under varying solution conditions. In addition, we have probed the ability of CE to detect insulin at physiological concentrations. This study is the first report of the use of UV-CE to monitor insulin oligomer formation at pH 8.0 and physiological ionic strength. Our results demonstrate the utility of CE as a complimentary technique for studying the early stages of insulin aggregation and define the hurdles that must be overcome before the aggregation of biological insulin concentrations can be explored.

## 2. Results and Discussion

### 2.1. Detection of Insulin Oligomers Using CE with UV Detection

**Figure 1:**
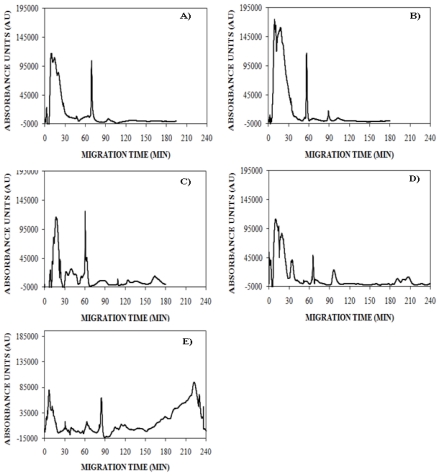
Detection of insulin monomer, oligomer, and higher molecular weight aggregation states using UV-CE. Insulin was aggregated under agitation (185 rpm) at 0.2 mg/mL in 40 mM Tris (pH 8.0) containing 150 mM NaCl and at 25 °C. At 0 h (panel **A**), 4 h (panel **B**), 8 h (panel **C**), 12 h (panel **D**), and 24 h (panel **E**), CE was performed in conjunction with UV detection with a 0.5 psi pressure injection for 8 s with separation at 15 kV using 0.5% PHEA separation matrix in a PHEA coated capillary. Results are representative of three independent experiments. Supplementary data confirms the absence of significant peaks at a migration time of >180 min for 0, 4, and 8 h time points.

To explore the use of CE for the detection of insulin oligomers that appear during early stages of insulin aggregation, lyophilized insulin was solubilized in 5 mM NaOH, diluted into 40 mM Tris (pH 8.0), subjected to 150 mM NaCl, and agitated at 185 rpm to promote amyloid assembly. The reaction was analyzed using UV-CE at early and late time points to assess the appearance of oligomers and progression into larger aggregate species. At 0 h, UV-CE demonstrated the presence of an early, broad peak in addition to a sharper peak migrating at ~70 min (Figure 1A). The size of these species was probed using a filtration analysis similar to that performed by Sabella *et al.* who used molecular weight cutoff membranes to size early amyloid-β aggregation species detected via UV-CE. For our experiments, we used membranes with molecular weight cutoffs of 30, 50, and 100 kDa to determine that the species present at 0 h correspond to molecular weights <30–50 kDa, or oligomers of <5–8 monomer units. A similar peak pattern was obtained after 4 h with the appearance of another peak migrating at ~90 min (Figure 1B). At 8 and 12 h, broad peaks migrating at times >90 min appeared (Figure 1C, D). The size of these species was estimated by filtration analysis to be >50 kDa, or larger than 8 monomer units, thus indicating the detection by UV-CE of the first species in the aggregation process. By 24 h, aggregate peaks of greater intensity appeared at migration times >150 min, indicating the formation of larger and more concentrated aggregate species, estimated via filtration analysis to be <100 kDa, or less than 17 monomer units (Figure 1E). Due to experimental time constraints, UV-CE runs for the 0, 4, and 8 h time points were terminated at 180 min. Separate experiments with run times of 240 min were conducted for the 0, 4, and 8 h time points and confirm that no significant species (signal to noise or S/N >3) were present at migration times >180 min (see supplementary materials).

Other measurement techniques have been employed previously to characterize insulin oligomers. Quasi elastic light scattering (QELS) [7], high performance liquid chromatography (HPLC) [53], small angle neutron scattering (SANS) [54], and nanoflow electrospray (nano-ES) mass spectrometry [55] have been successfully used to detect oligomeric insulin species. A study by Sluzky *et al.* utilized QELS to determine the particle diameter of insulin species generated upon agitation at 37 °C and 80 rpm in PBS (pH 7.4) [7]. Similar to the UV-CE results at 0 hr, a range of insulin species with diameters from 2.5 to 10 nm were observed initially in solution. Upon agitation for 1 h in the presence of Teflon spheres, a second peak appeared corresponding to insulin particles ~150 nm in diameter. After aggregation for 21 h, three species of insulin were present: native molecules with sizes ranging from 2.5 to 10 nm, stable intermediates with sizes ranging from 150 to 190 nm, and fully aggregated particles >800 nm. Nayak *et al.* and Vestergaard *et al*. utilized SANS and SAXS to monitor the formation of insulin oligomers and proposed a model for nucleus formation and growth [54,56]. However, insulin oligomers were generated under extreme conditions (45–65 °C, pH = 1.6–2.0, 5–10 mg/mL) which may not accurately reflect insulin aggregation *in vivo*. Nettleton *et al.* studied the time course of insulin oligomer appearance using nano-ES. Oligomers exhibiting sizes up to 12 monomeric units were detected when insulin was aggregated at very high, millimolar concentrations [55]; however, large aggregates could not be studied using this technique. In addition, identification of insulin oligomers was complicated by the presence of overlapping charge states among the aggregates present. The drawbacks of each technique listed above show that other complementary methods may be needed to verify the results obtained. The UV-CE method in the current study was able to detect insulin oligomers that appeared transiently during amyloid formation at a pH of 8.0 and at micromolar insulin concentrations. This highlights the potential for CE to be used as a complementary technique to follow the evolution of insulin oligomer appearance.

### 2.2. Effect of Salt Concentration on the Time Course for Insulin Oligomer Formation

**Figure 2:**
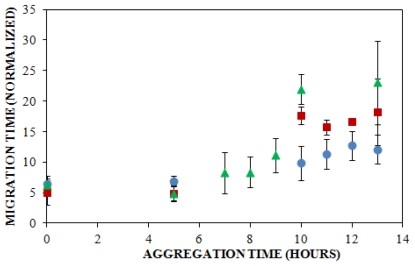
Effect of solution ionic strength on the formation of insulin oligomers detected by UV-CE. Insulin was diluted to 0.2 mg/mL in 40 mM Tris (pH 8.0) containing 100 mM ( 
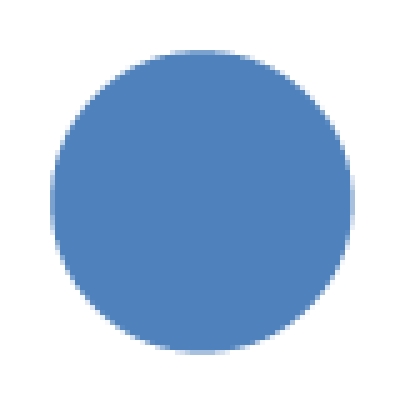
), 150 mM ( 
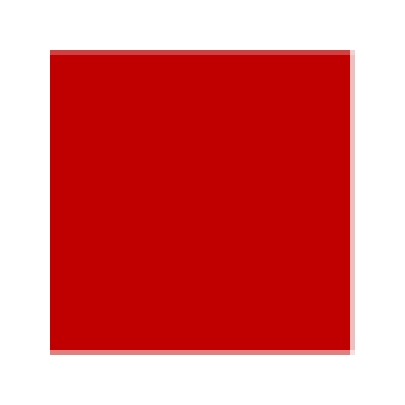
), or 250 mM ( 
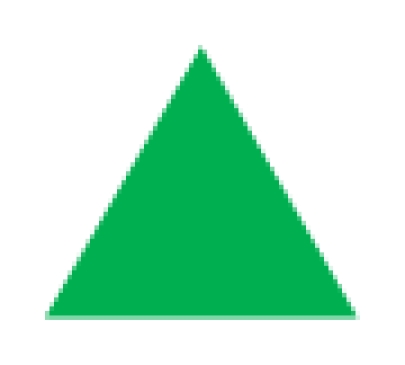
) NaCl. Aggregation was induced at 25 °C by continuous agitation (185 rpm) and monitored using UV-CE. CE was performed with sample injection at 0.5 psi for 8 s with 15 kV separation using 1% PHEA separation matrix in a PHEA coated capillary. Migration times were normalized to those observed prior to the onset of aggregation using the peak corresponding to monomer to facilitate comparison between individual runs. Error bars represent SE, *n* = 3. For the 10 h time point, the migration times of the 150 mM and 250 mM NaCl were both determined to be statistically different from the 100 mM NaCl migration time with a *p* < 0.1.

Solution conditions such as protein concentration [5,57], pH [5], and ionic strength [5,58,59] have been reported to have a pronounced impact upon the rate at which insulin aggregates, and understanding these effects can provide insight into the mechanism of insulin aggregation. Here, CE was employed to study the effect of solution ionic strength on the early events of insulin aggregation by examining the time to appearance of oligomers formed when insulin is aggregated at 25 °C and pH 8.0 (40 mM Tris) in the presence of three different concentrations of NaCl: 100 mM, 150 mM, and 250 mM. Figure 2 illustrates the change in normalized migration time of the largest species present throughout the early stages of aggregation. During the first 5 h of aggregation, there was little change in the migration time at all three salt concentrations. After 5 h, oligomeric species began to form. While the time to oligomer appearance was unaffected by NaCl concentration (Table 1), the size of oligomers formed increased with salt concentration. At 10 h, oligomers formed in the presence of 150 and 250 mM NaCl exhibiting significantly longer migration times than those formed in the presence of 100 mM NaCl (Figure 2). To our knowledge, no other studies have used methods focused on oligomer detection to examine the effect of ionic strength on insulin aggregation.

**Figure 3:**
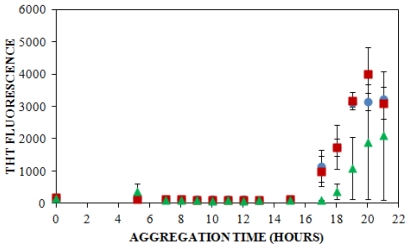
Effect of solution ionic strength on the formation of insulin aggregates detected by ThT binding. Insulin was diluted to 0.2 mg/mL in 40 mM Tris (pH 8.0) containing 100 mM ( 
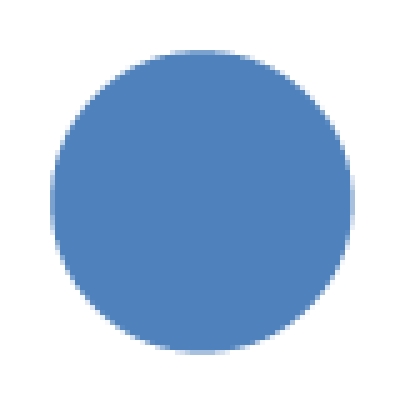
), 150 mM ( 
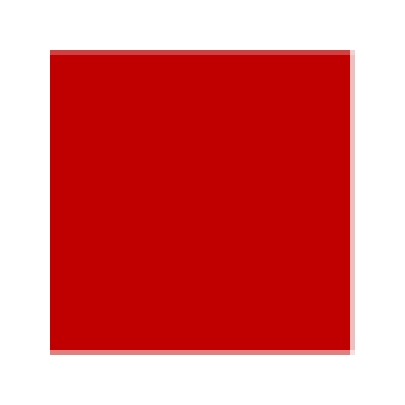
), or 250 mM ( 
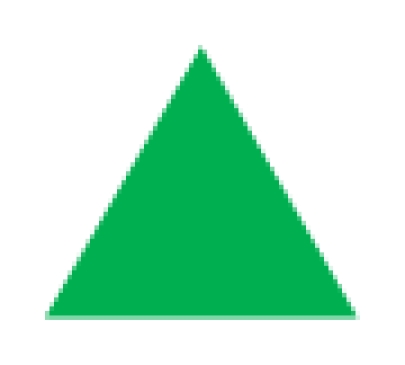
) NaCl. Aggregation was induced at 25 °C by continuous agitation (185 rpm) and monitored via ThT fluorescence by periodic dilution into 10 μM ThT. Error bars represent SE. Results are representative of two independent experiments.

A traditional method of detecting amyloid aggregates containing a cross β-sheet structure is through the examination of thioflavin T (ThT) binding, which has been used to study insulin aggregation under a variety of solution conditions. ThT is an intercalating fluorescent dye that binds to the β-sheet structure within amyloid fibrils, giving rise to a shifted excitation maximum at 450 nm and a shifted and enhanced emission at 482 nm [5,60]. For our study, ThT was also used to follow insulin aggregation in order to compare the lag times obtained using ThT fluorescence with those observed using CE. When insulin was aggregated at pH 8.0 (40 mM Tris) and 25 °C with agitation (185 rpm) in the presence of 100 mM NaCl, 150 mM NaCl, or 250 mM NaCl, the lag time, or initial increase in ThT fluorescence, was observed at 16 ± 1.0 h, at 15 ± 1.4 h, and at 19 ± 2.4 h, respectively (Figure 3, Table 1). These results demonstrate a trend toward a longer lag time at the highest salt concentration.

Other researchers have examined the effect of ionic strength on insulin structure [58,59] and aggregation lag time, but under slightly different conditions. At a similar solution pH of 7.0–8.0, lag times of 6–9 h have been reported in studies that have employed higher insulin concentrations [61] or higher temperatures with more vigorous agitation [62], which have both been reported to enhance amyloid protein aggregation [5,57,63–65]. Furthermore, changes in the lag time to ThT fluorescence have been observed to depend upon the change in solution ionic strength when insulin is aggregated under continuous agitation. Nielsen *et al.* observed that an increase in the NaCl concentration from 50 to 500 mM led to an initial decrease in the lag time from 1.6 to 1.3 h, whereas at the highest salt concentration of 500 mM, the lag time increased to 1.5 h [5]. Although much shorter lag times were observed is this study, likely due to the higher incubation temperature (37 °C) and acidic solution pH (1.6), the latter result parallels the effect of NaCl concentration on ThT detection of insulin aggregates observed in the current study, where an increase in the NaCl concentration to 250 mM resulted in an increase in the lag time (Figure 3).

**Figure 4:**
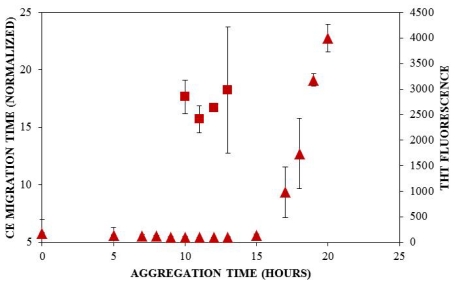
Comparison of lag times observed by UV-CE ( 
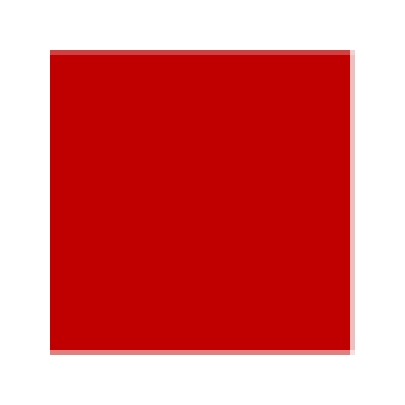
) and ThT binding ( 
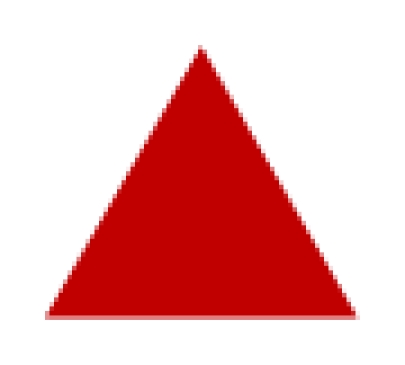
). Insulin was diluted to 0.2 mg/mL in 40 mM Tris (pH 8.0) containing 150 mM NaCl. Aggregation was induced at 25 °C by continuous agitation (185 rpm) and monitored via UV-CE or ThT fluorescence as described in Figure 2 and Figure 3, respectively.

When results from UV-CE (Figure 2) and ThT binding detection (Figure 3) of the initial aggregation state are compared (Figure 4, Table 1), it is clear that UV-CE is able to detect the aggregation process significantly earlier than ThT binding. In the presence of 100 and 150 mM NaCl, oligomers were detected using UV-CE 10 h prior to the observed increase in ThT fluorescence, and in the presence of 250 mM NaCl, UV-CE was able to resolve oligomers more than 11 h prior to the detection of aggregates using ThT. These differences most likely result from the inability of ThT to recognize early oligomeric species due to their lack of β-sheet structure. In contrast, UV-CE does not rely on the binding of a dye to this specific conformation but can instead detect oligomers regardless of their conformation. These results show that CE is capable of detecting early insulin oligomeric species while ThT binding can be used to verify the appearance of larger aggregates present in higher quantities. Therefore, CE and ThT binding can be used in a complimentary manner to detect species formed during all stages of aggregation.

Differences in detection capabilities of UV-CE and ThT binding lead to a more comprehensive understanding of the effect of NaCl on insulin aggregation. Results from UV-CE suggest that NaCl has little effect on the appearance of early aggregated states, shown by filtration studies to be oligomeric in nature. In contrast, results from ThT binding conversely suggest that increasing the NaCl concentration extends the lag time to formation of aggregated states with β-sheet conformations (Table 1). This comparison underscores the differences in amyloid protein aggregation that can be observed between oligomer and β-sheet aggregate behavior and emphasizes the need for a complimentary detection method, like CE, that can follow early stages in the aggregation process. A higher solution ionic strength could alter the structure of oligomers, leading to a slower conversion to the β-sheet structure detectable by ThT binding. Alternatively, concentrations of oligomers may remain low under conditions of higher solution ionic strength, thus precluding their detection by ThT binding, which exhibits high nanomolar to low micromolar limit of detection, for longer periods of time. An increase in the lag time detected by ThT binding at higher NaCl concentrations has also been observed in studies of other proteins that form amyloid aggregates [66]. The conclusion drawn by Lin *et al.* in these studies was that short and thick fibrils are formed at higher NaCl concentrations, and these fibrils are characterized by low intensity ThT binding signals. Thus, the ability of CE to detect insulin species independent of their conformation and at very low concentrations provides additional insight into the early events of insulin aggregation.

### 2.3. Determination of Insulin Limit of Detection

**Figure 5:**
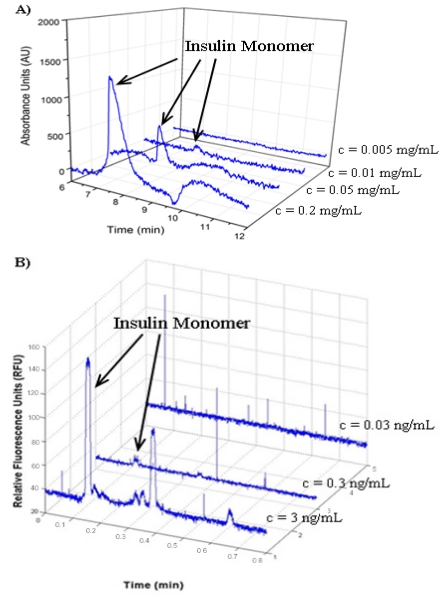
Limit of detection for monomeric insulin. (**A**) UV-CE detection of insulin with a 0.5 psi pressure injection for 8 s at 15 kV separation voltage using 0.1% PHEA separation matrix in a PHEA coated capillary; (**B**) LIF-CE detection of insulin with a 12 kV electrokinetic injection for 12 s at 15 kV separation voltage using 0.1% PHEA separation matrix in a PHEA coated capillary.

The ability to detect proteins at low concentrations will be necessary for the study of insulin aggregation at biological concentrations (300 pM) [67]. CE typically uses either UV absorbance or LIF to detect proteins. UV can detect proteins without any additional labeling, but typically has a lower sensitivity than LIF. LIF usually requires fluorescent labeling of the molecule to be detected, but is highly sensitive with previous reports of LIF-CE detection of double-stranded DNA down to the pg/μL range [68,69]. To determine the insulin detection limit using UV-CE, insulin monomer prepared at concentrations ranging from 0.005 mg/mL to 0.2 mg/mL was analyzed. The S/N ratio of the insulin peak was >3 at concentrations of 0.01 mg/mL and higher, defining 0.01 mg/mL (1.72 μM) as the limit of detection for insulin using UV-CE (Figure 5A). The definition of the detection limit as the analyte concentration with a S/N ratio >3 has been used previously in studies utilizing CE detection [70,71]. In addition, a similar limit of detection for insulin of 0.02 mg/mL (3.44 μM) has been obtained by Kunkel *et al.* using UV-CE [52].

A parallel limit of detection study was performed for fluorescein isothiocyanate (FITC)-labeled insulin monomer using LIF-CE. First, injection conditions were optimized by studying a range of sample injection voltages and injection times. As both the injection voltage and injection time were increased, observed insulin peaks increased in intensity. However, when the injection voltage and injection time were increased beyond 12 kV and 12 s, respectively, significant carryover of insulin between runs was observed due to the large amount of insulin injected into the capillary. Therefore, an injection voltage of 12 kV and an injection time of 12 s were selected as optimal. To determine the insulin detection limit using LIF-CE, FITC-labeled insulin monomer prepared at concentrations ranging from 0.03 to 3 ng/mL was analyzed. The S/N ratio of the insulin peak was >3 at concentrations of 0.3 ng/mL and higher, thus establishing 0.3 ng/mL (48.4 pM) as the limit of detection for FITC-labeled using LIF-CE (Figure 5B) and illustrating the superior limit of insulin detection for LIF-CE compared with UV-CE. In fact, the LIF detection limit of 48.4 pM is lower than the physiological insulin concentration of 300 pM [67] and to the authors’ knowledge, is the lowest LIF detection limit of insulin for an electrophoresis based method. Thus, LIF-CE is a promising technique for the detection of physiologically relevant insulin concentrations. Figure 5B also demonstrates the detection of four peaks in addition to the peak corresponding to monomeric protein. These additional peaks are most likely the presence of dimers and hexamers that have been reported to exist *in vitro* in freshly dissolved insulin solutions [72,73]. These species may be present at concentrations below the limit of insulin detection by UV-CE. The lower limit of detection offered by LIF-CE should facilitate the detection of these species.

### 2.4. Analysis of FITC Tracer Incorporation into Unlabeled Insulin

The ability of LIF-CE to detect insulin at sub-physiological concentrations suggests that this technique holds promise for the study of insulin aggregation at physiological insulin concentrations. Such studies will require the presence of a fluorescent label within insulin oligomers that appear during early stages of aggregation. Therefore, the ability of FITC-labeled insulin and unlabeled insulin to co-aggregate was explored. FITC-labeled insulin was selected because it has been previously shown to be an effective insulin label for LIF-CE applications [74,75]. FITC is covalently bound to the ɛ-amino groups of internal lysine residues and the α-amino group of the *N*-terminal residue. Unlike fluorescent amyloid-binding dyes, the covalent incorporation of this FITC label ensures its presence within both monomeric protein and aggregates that incorporate the labeled protein, including those that precede the appearance of β-sheet structure.

**Figure 6:**
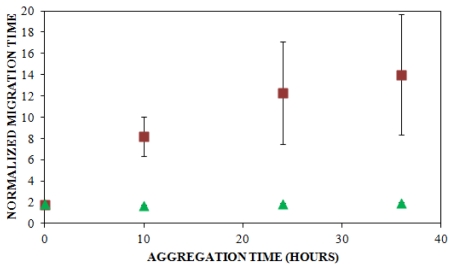
Coaggregation of FITC-labeled insulin and unlabeled insulin. Insulin solutions consisting of 25% FITC-labeled insulin and 75% unlabeled insulin with LIF detection ( 
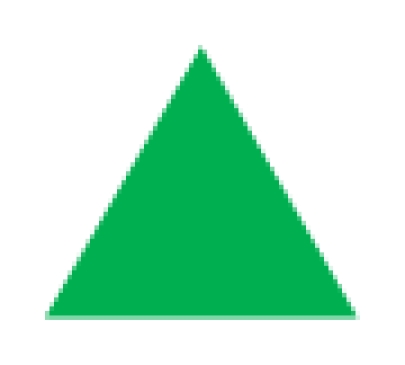
, *n* = 6) and UV detection ( 
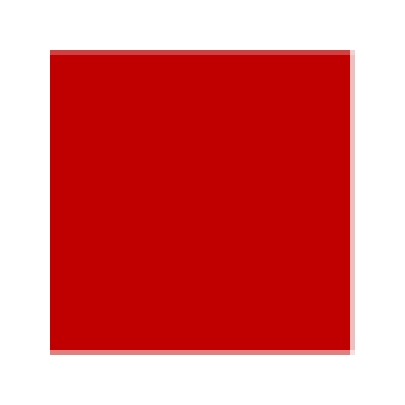
, *n* = 3) were prepared at a concentration of 0.2 mg/mL in 40 mM Tris (pH 8.0) containing 150 mM NaCl. Solutions were subjected to agitation (185 rpm), and the formation of aggregates was monitored. LIF-CE was performed with a sample injection at 12 kV for 12 s with 15 kV separation and UV-CE was performed with a sample injection at 10 kV for 12 s with 15 kV separation. Both separations were performed using 1% PHEA separation matrix in PHEA coated capillary. Migration times were normalized as described in Figure 2. Error bars represent SE, *n* = 3–6. Some error bars lie within symbols. For all time points >0 h, UV data was statistically different from the LIF data with a *p* < 0.015.

A sample consisting of 75% unlabeled insulin and 25% FITC-labeled insulin was prepared in 40 mM Tris (pH 8.0) containing 150 mM NaCl and agitated at 185 rpm to promote amyloid assembly. The reaction was analyzed using LIF-CE to assess the appearance of insulin oligomers. As shown in Figure 6, no change in the normalized migration time was observed over a 36 h period. Because oligomers of unlabeled insulin were observed using UV-CE beginning after 5 h following the onset of aggregation (Figure 2), this result suggested that the presence of the FITC label was preventing the aggregation of FITC-labeled insulin. To explore this possibility, LIF-CE was used to monitor the aggregation of 100% FITC-labeled insulin solubilized in Tris (pH 8.0), subjected to 150 mM NaCl, and agitated at 185 rpm (data not shown). Again, the normalized migration time was unchanged during the first 24 h following the initiation of agitation, confirming that the presence of the FITC label prevents aggregation within this timeframe.

To further determine whether the FITC-labeled insulin was inhibiting the formation of unlabeled insulin aggregates or failing to incorporate into aggregates formed from the unlabeled protein, UV-CE was performed in parallel with LIF-CE to monitor the aggregation of 75% unlabeled insulin and 25% FITC-labeled insulin solubilized in 40 mM Tris (pH 8.0) containing 150 mM NaCl and subjected to agitation at 185 rpm. As shown in Figure 6, the normalized migration time for UV-CE increased significantly over a period of 36 h, beginning by 10 h following the onset of agitation, while the normalized migration time for LIF-CE remained unchanged. These results indicate that insulin oligomers and larger aggregates were formed from the unlabeled protein and that the FITC-labeled insulin did not incorporate into these aggregates. Since some small compounds have been previously reported as inhibitors of β-sheet formation, it is possible that the FITC label is acting as an inhibitor to insulin aggregation. Another possibility is that the FITC attachment site is critical for proper β-sheet folding. A similar extension of the lag time to aggregation has been observed following the methylation of amino groups within the amyloid-β protein [76] and the introduction of a mutant that mimics phosphorlyation of serine residues within Huntington protein [77]. In addition, the quantity of amyloid aggregates formed is reduced following the citraconylation of lysine residues within lysozyme [78] or stilbine modification of ɛ-amino groups within transthyretin [79]. Therefore, dyes with alternative properties or attachment sites need to be explored. In particular, less bulky fluorescent probes, such as BODIPY, or attachment of dyes exclusively at the *N*- or *C*- terminus would be less likely to impact aggregate formation.

## 3. Materials and Methods

### 3.1. Materials

Previous studies have shown no differences between the three-dimensional structures of bovine and synthetic human insulin [80] and the binding affinity of bovine and synthetic insulin to insulin receptors at three major sites of insulin action are similar [81]. Similar to human insulin, bovine insulin contains 51 amino acids but differs from human insulin in residues A8 (Thr→Ala), A10 (Iso→Val), and B30 (Thr→Ala) [82]. Therefore, bovine insulin was used for all studies. Insulin and fluorescein isothiocyanate (FITC)-labeled insulin from bovine pancreas, poly-*N*-hydroxyethyl acrylamide (PHEA) and thioflavin T (ThT) were obtained from Sigma-Aldrich (St. Louis, MO). Polymerization initiation compound 2,2′-azobis(2-amidinopropane) dihydrochloride (V-50) was purchased from Wako Chemical (Richmond, CA). Amicon centrifugal filter units were purchased from Millipore (Billerica, MA).

### 3.2. Insulin Preparation

Lyophilized insulin and FITC-labeled insulin were stored at −20 °C. Unlabeled insulin was reconstituted to a final concentration of 0.005–0.2 mg/mL in 40 mM Tris (pH 8.0) containing 0–250 mM NaCl. FITC-labeled insulin was reconstituted to a final concentration of 0.03 ng/mL–0.2 mg/mL in 40 mM Tris (pH 8.0) containing 0–150 mM NaCl. Samples consisting of 75% unlabeled insulin and 25% FITC-labeled insulin were prepared by mixing the necessary proportions of insulin and FITC-labeled insulin from individual stock concentrations of 0.3 and 0.2 mg/mL, respectively.

### 3.3. Electrophoresis Conditions for UV and LIF Studies

All studies were carried out in 0.1% w/v PHEA coated capillaries with a 0.1–1% PHEA separation matrix and a capillary temperature of 25 °C. Capillary dimensions for UV-CE studies were *L*_t_ = 31 cm, *L*_d_ = 10 cm and for LIF-CE studies were *L*_t_ = 36 cm, *L*_d_ = 10 cm. The first UV-CE study was conducted using a 0.5% PHEA separation matrix. For the study on the effect of salt concentration on insulin oligomer formation, the capillary was filled with 1% PHEA and rinsed with 40 mM Tris (pH 8.0) for 5 min prior to each run. This rinse was utilized to dilute the PHEA on-column and overcome the long run times associated with the 0.5% PHEA separation matrix. Polymers of HEA were synthesized as described previously [83] with the following changes: 4% w/w initial monomer concentration and polymerization for 5 h. CE separations using UV detection were carried out using a P/ACE MDQ Glycoprotein System from Beckman Coulter, Inc. (Fullerton, CA) (214 nm filter) interfaced with an IBM computer utilizing 32 Karat software (V. 5.0, Beckman Coulter, Inc.) for data collection. Samples were pressure injected at 0.5 psi for 8 s and separated at 15 kV. Between each run, the capillary was rinsed with deionized water for 10 min to ensure that the insulin was not retained on the capillary wall. CE separations using LIF detection were carried out using an Applied Biosystems (Foster City, CA) 3130 Genetic Analyzer (excitation = 494 nm, emission = 522 nm) interfaced with a Dell computer utilizing Foundation Data Collection V 3.0 software. Samples were electrokinetically injected at 10 or 12 kV for 12 s and separated at 15 kV.

### 3.4. Limit of Detection Studies

Unlabeled insulin was prepared at concentrations of 0.05–0.2 mg/mL in 40 mM Tris (pH 8.0), and FITC-labeled insulin was prepared at concentrations of 0.03–3 ng/mL. Immediately following preparation, 100 μL samples of unlabeled insulin and 10 μL samples of FITC-labeled insulin were analyzed by UV-CE or LIF-CE, respectively, to determine the intensity of the first peak. Between runs for determining the limit of detection, the capillary was rinsed with deionized water for 20–120 min, and elution of 40 mM Tris (pH 8.0) was analyzed to ensure that insulin was not retained on the capillary wall.

### 3.5. Oligomer Formation Assay

To observe the time course for insulin oligomer formation, insulin was solubilized in 5 mM NaOH for 30 min and diluted into 40 mM Tris (pH 8.0) to make a 1 mg/mL stock. The stock was then diluted to 0.2 mg/mL in 40 mM Tris (pH 8.0) containing 150 mM NaCl and incubated at 25 °C under continuous agitation (185 rpm). At times of 0, 4, 8, 12, and 24 h, a 50 μL sample was removed and analyzed by UV-CE, with 0.5% PHEA separation matrix, to determine the migration time and intensity of all peaks. Separate experiments were conducted using the same sample preparation and CE conditions in order to determine the size range of insulin oligomers observed. At 0, 4, 8, and 12 h, a 50 μL sample was taken and ultrafiltrated (20 min, 14,000 × *g*) through Amicon filters with cut-off values of 30 kDa, 50 kDa and 100 kDa. The filtrate was removed and analyzed via UV-CE to determine the relative size of oligomers.

To examine the effect of solution ionic strength on insulin oligomer formation, insulin was prepared at a concentration of 0.2 mg/mL in 40 mM Tris (pH 8.0) containing 100, 150, or 250 mM NaCl. Samples were incubated at 25 °C under continuous agitation (185 rpm). Both prior to the onset of agitation and at times between 5 and 24 h following the onset of agitation, a 50 μL sample was removed and analyzed by UV-CE to determine the migration time of the first and last peaks. In parallel experiments, aggregation was monitored using ThT binding as described previously [84] by diluting an aliquot into ThT (10 μM) and evaluating fluorescence using a Perkin-Elmer LS-45 luminescence spectrometer (Waltham, MA) (excitation = 450 nm, emission = 470–500 nm) with baseline (ThT) subtraction. Lag times to aggregate formation were determined for individual runs as the last time point prior to a marked increase in signal. For UV-CE, this increase was an extension of the migration time for the last peak greater than 2-fold that of the monomer migration time. For ThT binding, this increase was 5% of the fluorescence observed at equilibrium.

The co-incorporation of unlabeled insulin and FITC-labeled insulin into oligomers was examined using LIF-CE and UV-CE in parallel. FITC-labeled insulin was prepared alone at a concentration of 0.2 mg/mL or combined with unlabeled insulin for final concentrations of 0.2 mg/mL unlabeled insulin and 0.067 mg/mL FITC-labeled insulin (75% unlabeled, 25% FITC-labeled). Both samples were prepared in 40 mM Tris (pH 8.0) containing 150 mM NaCl and incubated at 25 °C under continuous agitation (185 rpm). Both prior to the onset of agitation and at times between 5 and 24 h following the onset of agitation, the migration times of the first and last peak were determined by both UV-CE and LIF-CE. Here, a 50 μL sample was removed for analysis by UV-CE and a 20 μL sample was removed and diluted to 0.013 mg/mL for analysis by LIF-CE.

### 3.6. Statistical Analysis

The migration time and intensity of peaks were analyzed using Chromagna (VO 9.8) software (provided by Mark Miller, NIH) and Origin (V. 8.0) software from OriginLab Corporation (Northampton, MA). Chromagna software was used to convert the fsa file format of the ABI 3130 Genetic Analyzer to excel files, which are compatible with Origin. A Gaussian fit was used to calculate the peak area and migration time in Origin. The migration times of peaks observed in the insulin oligomer time course and salt concentration studies were normalized in order to draw qualitative conclusions about the sizes of insulin species present at various times throughout aggregation. Peak migration times were determined by normalizing the migration time for the last peak observed relative to the migration time of the first peak observed prior to the onset of aggregation. In addition, the peak height for the monomeric peaks detected in the UV and LIF limit of detection studies was determined and the S/N ratio was calculated. Peaks with a S/N ratio >3 were considered significant. Statistical analysis for comparison of lag times was performed using Prism 5 software (GraphPad Software Inc., San Diego, CA). The effect of detection method upon lag time was assessed using a one-way ANOVA with Bonferroni post-test. Unpaired *t*-tests were performed using GraphPad QuickCalcs (GraphPad Software Inc., San Diego, CA) to compare CE normalized migration times.

## 4. Conclusion

Insulin aggregation poses problems both *in vivo* and *in vitro*. These problems include injection site bleeding and bruising which can occur during the treatment of Type II diabetes in addition to problems with the pharmaceutical quality control of insulin. Elucidating the molecular mechanism by which insulin aggregation occurs, in particular the early stages of aggregation during which oligomeric species are formed, will facilitate the prevention of these problems. However, most techniques utilized for studies of insulin aggregation are not sensitive enough to detect physiologically relevant concentrations or oligomeric species present transiently throughout aggregation under physiologically relevant solution conditions. These limitations highlight the importance of employing a complementary technique to explore the evolution of insulin oligomer appearance at physiologically relevant concentrations. The current study illustrates that CE is a promising technique for monitoring the appearance of oligomeric species during the early events of insulin aggregation and is the first report of the use of UV-CE to monitor insulin oligomer formation at pH 8.0 and physiological salt concentration. UV-CE was employed to demonstrate that a change in salt concentration from 100 mM NaCl to 250 mM NaCl had little effect on the formation of small oligomeric species. A comparison between the use of UV-CE and ThT binding for monitoring insulin aggregation revealed that CE was able to detect the appearance of aggregated species at significantly earlier times than ThT binding, demonstrating that CE and ThT binding may be used as complementary techniques to identify insulin species present at all times throughout aggregation. The lowest concentration of monomeric insulin that can be detected was determined using both UV and LIF detection modes. Physiologically relevant insulin concentrations in the picomolar range were detectable using LIF detection while concentrations in the micromolar range were required for UV detection. Using UV-CE and LIF-CE to simultaneously monitor the aggregation of a mixture of FITC-labeled insulin and unlabeled insulin, this study was the first to show that FITC-labeled insulin was unable to incorporate into oligomers formed by the unlabeled protein. These results demonstrate that while CE is a promising technique for the detection of physiologically relevant insulin concentrations, caution must be taken when choosing a dye for detection of oligomeric and aggregate insulin species. This necessitates further investigation to identify optimum fluorescent labels for the study of insulin oligomer formation at physiological insulin concentrations.

## Supplementary Material



## Figures and Tables

**Table 1:** Lag times observed at 100, 150, and 250 mM NaCl by CE *versus* ThT binding.

NaCl concentration (mM)	Lag time for CE (h) 1	Lag time for ThT Binding (h) 2
100	6.7 ± 1.7	16 ± 1.0 ***
150	5.0 ± 0.0	15 ± 1.4 ***
250	7.6 ± 1.3	19 ± 2.4 ***

1 Results are reported as the mean ± SE, *n* = 3.

2 Results are reported as the mean ± SE, *n* = 4.

*** *p* < 0.001 for comparison between detection via UV-CE and ThT binding.
