# Supplementary material for: Monitoring Insulin Aggregation via Capillary Electrophoresis
Source: Int J Mol Sci. 2011 Dec 14;12(12):9369–88. doi: 10.3390/ijms12129369 (PMC3257135; doi:10.3390/ijms12129369)

Supplementary Information

Figure S1. Detection of insulin oligomeric species using UV-CE. Insulin was aggregated under agitation (185 rpm) at 0.2 mg/mL in 40 mM Tris (pH 8.0) containing 150 mM NaCl and at 25 °C. At 0 h (panel **A** and **B**), 4 h (panel **C** and **D**), and 8 h (panel **E** and **F**), CE was performed in conjunction with UV detection with a 0.5 psi pressure injection for 8 s with separation at 15 kV using 0.5% PHEA separation matrix in a PHEA coated capillary, *n* = 3. S/N ratios for peaks beyond 180 min are given at 0 h (panel **B**) and 8 h (panel **F**).

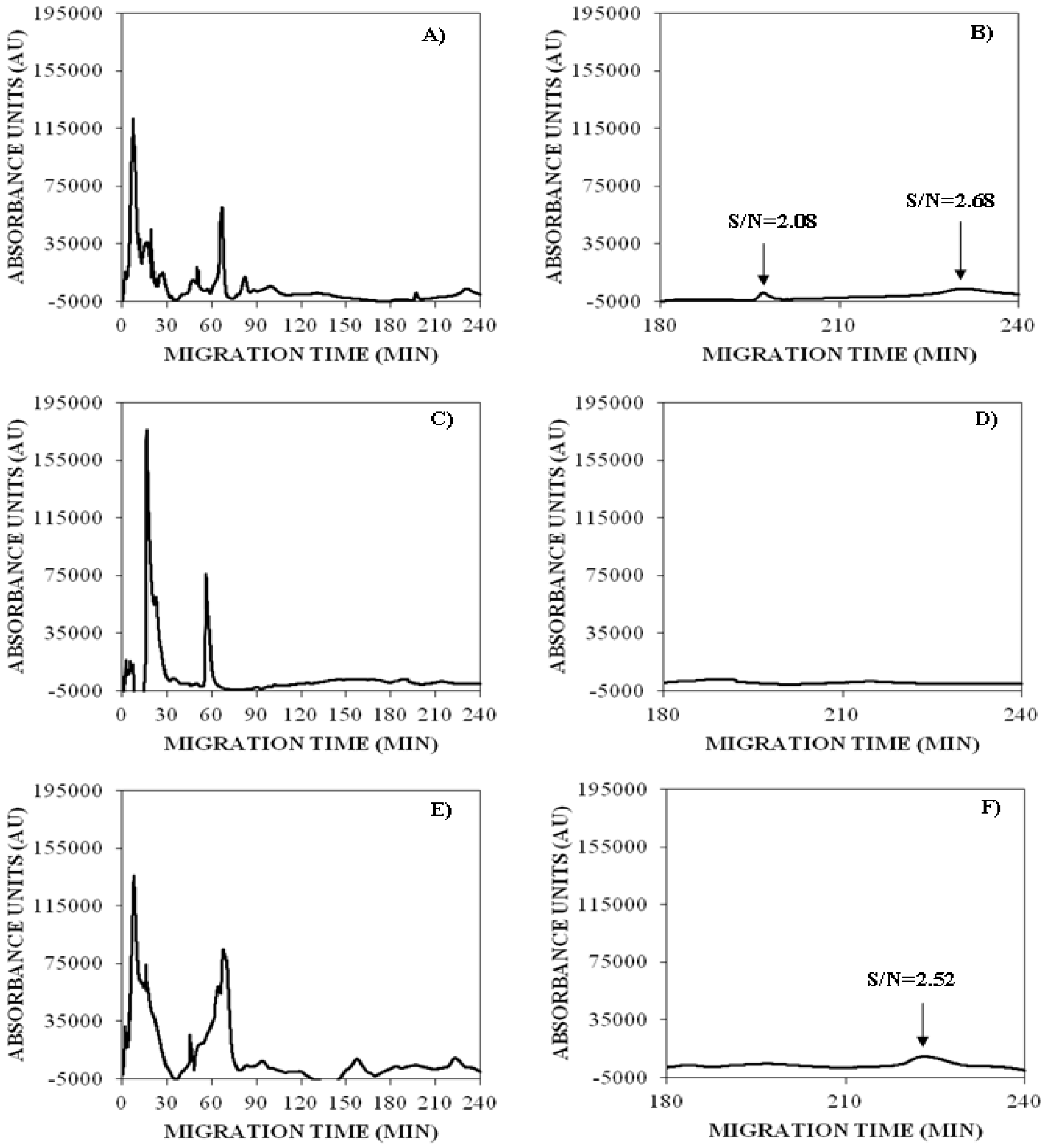

Supplement: Supplementary file 1 [file ijms-12-09369-s001.pdf]
